# Therapeutically targeting essential metabolites to improve immunometabolism manipulation after liver transplantation for hepatocellular carcinoma

**DOI:** 10.3389/fimmu.2023.1211126

**Published:** 2023-07-10

**Authors:** Wenhui Zhang, Yu Zhao, Qiang He, Ren Lang

**Affiliations:** ^1^ Department of Hepatobiliary Surgery, Beijing Chao-Yang Hospital Affiliated to Capital Medical University, Beijing, China; ^2^ Department of Urology Surgery, Beijing Chao-Yang Hospital Affiliated to Capital Medical University, Beijing, China

**Keywords:** hepatocellular carcinoma (HCC), liver transplantation (LT), ischemia-reperfusion (IR) injury, succinate, immunometabolism, lipid metabolism

## Abstract

Hepatocellular carcinoma (HCC) is the most prevalent primary liver malignancy worldwide and is associated with a poor prognosis. Sophisticated molecular mechanisms and biological characteristics need to be explored to gain a better understanding of HCC. The role of metabolites in cancer immunometabolism has been widely recognized as a hallmark of cancer in the tumor microenvironment (TME). Recent studies have focused on metabolites that are derived from carbohydrate, lipid, and protein metabolism, because alterations in these may contribute to HCC progression, ischemia-reperfusion (IR) injury during liver transplantation (LT), and post-LT rejection. Immune cells play a central role in the HCC microenvironment and the duration of IR or rejection. They shape immune responses through metabolite modifications and by engaging in complex crosstalk with tumor cells. A growing number of publications suggest that immune cell functions in the TME are closely linked to metabolic changes. In this review, we summarize recent findings on the primary metabolites in the TME and post-LT metabolism and relate these studies to HCC development, IR injury, and post-LT rejection. Our understanding of aberrant metabolism and metabolite targeting based on regulatory metabolic pathways may provide a novel strategy to enhance immunometabolism manipulation by reprogramming cell metabolism.

## 1 Introduction

Hepatocellular carcinoma (HCC) has become the second leading cause of cancer-related deaths worldwide, with a large number of patients diagnosed each year (1). Several treatment strategies are available for HCC, such as surgical resection or liver transplantation (LT), transcatheter arterial chemoembolization (TACE), selective internal radiation therapy (SIRT), chemotherapy, and immunotherapy (2, 3). However, although LT is the most fundamental and effective treatment for non-end-stage HCC, mortality and recurrence rates remain high. Additionally, ischemia-reperfusion (IR) injury and rejection after LT may severely impair graft function. Therefore, it is necessary to further elucidate the characteristics of HCC and the possible mechanisms of IR injury and rejection and to develop novel therapies. The duration of IR and the tumor microenvironment (TME), which may be hypoxic, acidic, and deficient in nutrients, can induce changes in tumor cell metabolism and neighboring stromal cells, such as tumor-associated macrophages (TAMs), dendritic cells (DCs), and lymphocytes, thereby promoting tumor survival, proliferation, and invasion (4). Recently, various metabolic abnormalities have been identified in the TME of HCC, IR, and rejection during LT, with aberrant metabolism being an area that has garnered significant attention in recent years. Dysregulation of cancer metabolism, IR injury, and rejection, particularly succinate metabolism, in which oncogenic signaling pathways are aberrantly activated, altering the expression and activity of metabolic enzymes, has been considered a fundamental metabolic rewiring phenomenon in tumor cells and immunocytes. Furthermore, it may also be involved in the development and progression of HCC and IR injury or rejection. This review aims to address how dysregulation models HCC cells and neighboring immunocytes, supports HCC progression, IR injury, and rejection, and explores specific ways to therapeutically target metabolites to treat HCC patients, reverse IR injury, and achieve immune tolerance.

## 2 Targets in carbohydrate metabolism

Targeting immune checkpoint inhibitors (ICIs) has been recently shown to have a significant overall survival (OS) benefit in patients with HCC (5). Targeting metabolites may also be a promising approach for the treatment of HCC or recurrence after LT. Metabolites are intermediates in carbohydrate metabolism that play a regulatory role in cell physiology, such as tumorigenesis, proliferation, invasion, metastasis, and chemoresistance. Different altered metabolic pathways, involving metabolites as either oncogenes or tumor suppressors, mediate the molecular pathogenesis of HCC. Among the developed biomarkers, metabolites such as succinate, acetate, and itaconate are particularly promising due to their unique chemical properties. Recent studies suggest that metabolites may be promising biomarkers for HCC treatment. This review provides an overview of the metabolic pathways of succinate/succinate dehydrogenase (SDH), acetate, and itaconate in the context of cancer metabolism and IR injury during transplantation. The primary objective is to identify potential targets for mitigating IR injuries and combating cancer.

### 2.1 Succinate modifications in cancer-immune cross-talk

**Figure 1:**
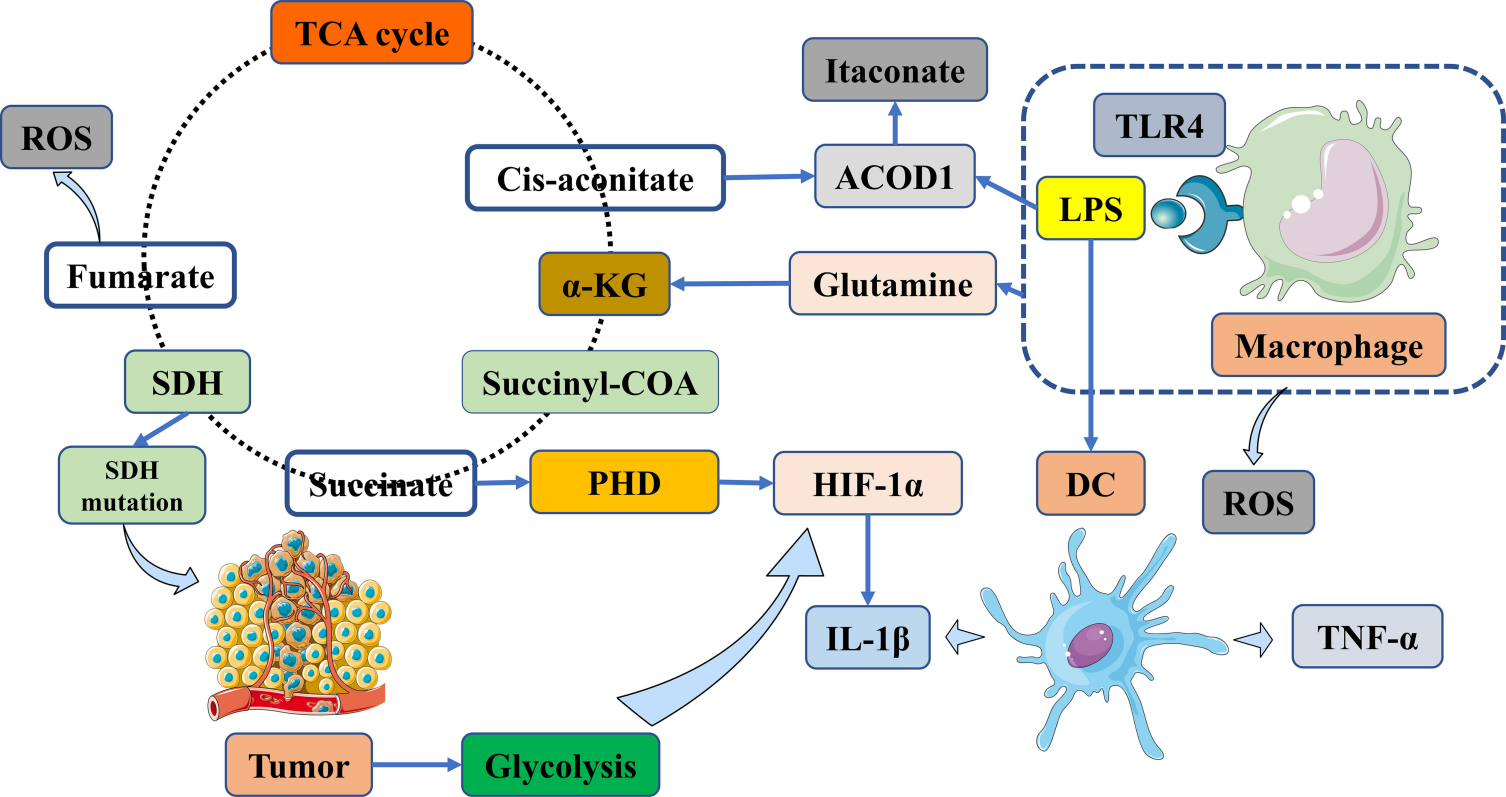
Targeting vital metabolites as promising therapeutic targets via immunometabolism manipulation. Tumors are biased towards aerobic glycolysis in the TME. Macrophages produce ROS in addition to TNF-α and IL-1β. Subsequently, succinate accumulation stabilizes HIF-1α via the inhibition of PHD enzyme activity and the generation of IL-1β. Succinate accumulation results from TLR4 signaling and LPS-activated macrophages via glutamine metabolism. Itaconate is synthesized from cis-aconitate in the TCA cycle in macrophages, which is activated by LPS and other TRL ligands; these stimuli improve ACOD1, which then shifts cis-aconitate out of the TCA cycle to produce aconitate. TME, tumor microenvironment; ACOD1, aconitate decarboxylase 1; α-KG, α-ketoglutarate; DC, dendritic cell; HIF-1α, hypoxia-inducible factor-1 alpha; LPS, lipopolysaccharide; PHD, prolyl hydroxylase domain; ROS, reactive oxygen species; SDH, succinate dehydrogenase; TCA cycle, tricarboxylic acid cycle; TLR, toll-like receptor; TNF-α, tumor necrosis factor alpha; IL-1β, interleukin-1 beta.

Recent experimental evidence has shown that succinate plays multiple roles in immunity and cancer (6–8). Several studies have suggested that succinate regulates tumorigenesis in specific microenvironments (9). Succinate is often considered a traditional signal for tumorigenesis (10). However, modifications in cancer-immune cross-talk indicate that SDH may act as a tumor suppressor. According to experimental data, SDH mutations can lead to succinate accumulation and subsequent stabilization of hypoxia-inducible factor-1 alpha (HIF-1α), thereby promoting tumor growth (11) (**Figure 1**). M1 macrophages can produce reactive oxygen species (ROS) and inflammatory cytokines, such as tumor necrosis factor-alpha (TNF-α) and interleukin-1 beta (IL-1β), which contribute to metabolic alterations and cancer-related inflammation (12) (**Figure 1**).

Numerous studies have shown that lipopolysaccharide (LPS)-activated macrophages can lead to succinate accumulation through inhibition of the prolyl hydroxylase domain (PHD), which further stabilizes HIF-1α, as supported by experimental evidence (**Figure 1**). HIF-1α, coupled with the expression of target genes encoding glycolytic enzymes and the pro-inflammatory cytokine IL-1β, can block tumor growth and survival and exacerbate inflammation (13–15) (**Figure 1**). Targeting the HIF-1α pathway is a promising strategy, as it plays a crucial role in attenuating liver IR injury (16). Experimental evidence has shown that the loss of activating transcription factor 3 (ATF3) exacerbates liver damage by activating the mammalian target of rapamycin (mTOR)/p70S6K/HIF-1α signaling pathway in inflammatory liver injury. Furthermore, Wang et al. have shown that deficiency of the plasma membrane-bound G protein-coupled bile acid receptor (TGR5) exacerbates hepatic IR injury by inhibiting the sirtuin 3 (SIRT3)/forkhead box O3 (FOXO3)/HIF-1α pathway (17). Therefore, current studies are identifying potential therapeutic targets via the HIF-1α pathway for the treatment of liver IR injury following LT (18). In summary, succinate may have a dual role in tumors, either promoting or inhibiting tumor growth, whereas SDH has only been identified as playing a role in suppressing tumor growth and progression.

In the context of cancer-immune cross-talk, succinate can also affect immune cells, namely antigen-presenting cells (APCs) and T cells, suggesting that targeting succinate may be a viable approach to modulating immune responses. Succinate has been shown to enhance the antigen-presenting capacity of APCs and induce an adaptive immune response that can block tumor growth, particularly in DCs (19). When stimulated by antigens, a significant increase in antigen-specific T cell activation can promote the production of cytokines such as TNF-α and interferon-gamma (IFN-γ) during immune activation (20). These cytokines can kill cancer cells and prolong the survival of patients with tumors (21, 22).

### 2.2 Regulatory mechanisms of succinate and SDH

Succinate and SDH are critical for energy supply in the tricarboxylic acid (TCA) cycle of conventional metabolism. Succinate is produced by the catalysis of succinyl-CoA by succinyl-CoA synthetase (**Figure 1**). Immediately after the generation of succinate, it undergoes fumarate production catalyzed by SDH, accompanied by a burst of reactive oxygen species (ROS) (**Figure 1**). SDH is a complex consisting of four nuclear-encoded subunits. However, it is still unclear whether succinate and SDH are modulated in HCC, in contrast to a subset of cancers, especially neuroendocrine tumors, caused by SDH mutations (**Figure 1**).

**Figure 2:**
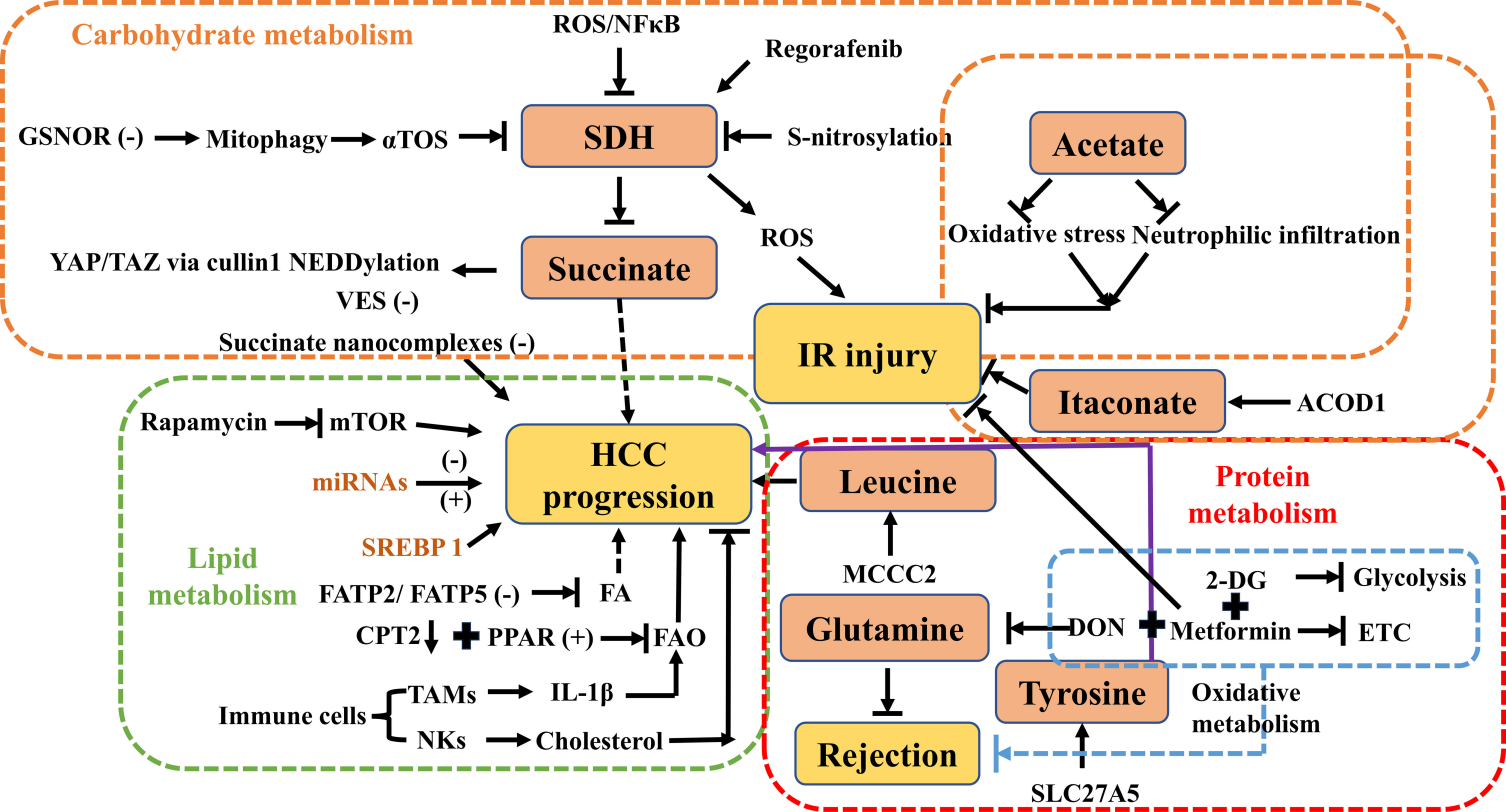
Overview of carbohydrate, lipid, and protein metabolism by targeting central metabolites in HCC, IR injury, and rejection. ROS, reactive oxygen species; GSNOR, denitrosylating enzyme S-nitrosoglutathione reductase; αTOS, alpha-tocopheryl succinate; SDH, succinate dehydrogenase; YAP/TAZ, Yes-associated protein/transcriptional coactivator with PDZ-binding motif; VES, vitamin E succinate; IR injury, ischemia-reperfusion injury; ACOD1, aconitate decarboxylase 1; HCC, hepatocellular carcinoma; mTOR, the mammalian target of rapamycin; miRNA, microRNAs; SREBP-1, sterol regulatory element‐binding protein 1; FA, fatty acid; FAO, fatty acid oxidation; FATP, fatty acid transport protein; TAMs, tumor‐associated macrophages; IL-1β, interleukin-1 beta; NKs, natural killer cells; MCCC2, methylcrotonoyl-CoA carboxylase 2; DON, 6-diazo-5-oxo-L-norleucine; SLC27A5, the solute carrier family 27 member 5; ETC, electron transport chain; CPT2, carnitine palmitoyltransferase 2; 2-DG, 2-deoxy-D-glucose.

Clinical data have shown that decreased SDH in combination with elevated succinate is associated with a poor prognosis in patients with HCC (23). In a recent biomedical study, it was revealed that SDH reduction promotes HCC proliferation by impairing the proteasomal degradation of Yes-associated protein (YAP)/transcriptional coactivator with PDZ-binding motif (TAZ) through regulation of cullin1 NEDDylation (**Figure 2**). Based on these findings, targeting succinate and SDH under the regulation of YAP/TAZ may provide potential therapeutic strategies for HCC (23) (**Figure 2**). According to research findings, aberrant S-nitrosylation sensitizes tumor cells to SDH inhibition in HCC, providing a possible molecular target in SDH for the treatment of liver cancer (24) (**Figure 2**). Similarly, mitophagy inhibition via the denitrosylating enzyme S-nitrosoglutathione reductase (GSNOR) leads to antitumor effects of the mitochondrial complex II inhibitor alpha-tocopheryl succinate (αTOS) (25) (**Figure 2**). From a clinical translation perspective, a polymer of vitamin E succinate (VES) has been designed to target tumors and enhance antitumor activity (26) (**Figure 2**). Many researchers have developed appropriate drug delivery systems targeting HCC cells to inhibit tumor growth and improve the efficacy of chemotherapeutic agents, even by overcoming resistance (27–32). A recent paper suggests that SDH knockdown to inactivation promotes HCC growth and metastasis via ROS/NFκB signaling (33) (**Figure 2**). As a key tumor suppressor metabolite, SDH provides strong evidence to support the targeting of succinate for the treatment of HCC. Regorafenib, a clinically targeted drug, plays a role in inducing cell apoptosis and promoting SDHD expression (**Figure 2**). SDHD is associated with cell proliferation and the therapeutic effect of regorafenib. This suggests that SDHD may be a potential drug target for HCC (34). Succinate nanocomplexes may be an effective strategy for the treatment of drug-resistant HCC based on clinical research (35) (**Figure 2**).

In conclusion, SDH mutations are related to the Warburg effect, also known as aerobic glycolysis, which shapes immune responses to inhibit tumor cell dedifferentiation, proliferation, and invasion in patients with HCC. Polydrugs have been designed with succinate to reverse resistance to chemotherapeutic agents. These findings suggest that targeting succinate and SDH may be a promising therapeutic strategy for HCC treatment, and further research in this area may lead to the development of more effective treatments for this disease.

### 2.3 Targeting succinate metabolism in IR injury

IR injury is a critical factor for graft survival in organ transplantation. It is well-known that IR injury is one of the main causes of primary graft dysfunction after LT, and approximately 10% of donor livers fail due to IR injury, which also leads to a high incidence of acute and chronic rejection. Prevention of graft dysfunction may be achieved by developing new metabolites based on recent research that has implicated mitochondrial succinate metabolism in IR injury. Understanding the role of succinate and SDH in IR injury may provide insight into potential therapeutic targets for the prevention and treatment of graft dysfunction in LT. Developing new metabolites that target succinate and SDH may be a promising approach to improving graft survival and reducing the incidence of acute and chronic rejection following LT. Further research in this area is needed to identify and develop effective therapies for IR injuries in the context of LT.

Although the underlying mechanism of IR injury remains unclear, ROS production is central to IR injury in reperfused ischemic tissue - this provides new therapeutic approaches within the mitochondria as a whole (36) (**Figure 2**). Succinate is increased in the TCA cycle or the citric acid cycle during ischemia, and it can be catalyzed by SDH, resulting in a corresponding ROS burst during reperfusion. This, in turn, leads to calcium dysregulation and ATP depletion, which subsequently result in reperfusion injury (37). Targeting succinate metabolism opens up an impressive therapeutic strategy. Recent studies have reported that malonate can reduce IR injury during ischemia or upon reperfusion by inhibiting SDH, which is a key enzyme in cardioprotective effects *in vivo* (6). A possible explanation is that disruption of both succinate accumulation in ischemia and oxidation in reperfusion alters succinate metabolism (6). Succinate is selectively oxidized by mitochondria to accumulate in chronic heart failure, ischemic stroke, and renal IR injury. IR injury is mitigated by malonate therapy, which is a promising therapeutic application during LT. Disruption of succinate metabolism and modulation of SDH bridge the gap between IR injury and LT. Although many metabolites have shown promise in preventing IR injury *in vitro* and *in vivo*, translational studies have proven to be a long road from bench to bedside (6, 36).

The main technical difficulty in donor liver preservation stems, as it should, from succinate accumulation. As cold ischemia time increases during static cold storage (SCS), intracellular ATP is gradually depleted, causing mitochondrial complex II dysfunction and interruption of aerobic respiration, leading to succinate accumulation. Succinate accumulation aggravates mitochondrial complex I dysfunction, which further releases ROS and results in irreversible graft injury. Hypothermic oxygenated machine perfusion (HOPE) presents a significant improvement in recipient outcomes in ROS release and ATP depletion compared to SCS (38, 39).

### 2.4 Acetate and itaconate for the manipulation of immunometabolism in LT

In addition to succinate, acetate and itaconate provide targeted signals to regulate immunometabolism, contribute to the manipulation of IR injury, and induce immune tolerance. Acetate has explosive growth toward glycolysis in metabolic reprogramming during T cell-mediated responses (40). Glatiramer acetate, a mixture of four amino acids, has been shown to have multiple roles in immunomodulation and anti-inflammatory effects for the treatment of multiple sclerosis (MS). Surprisingly, a new comprehensive study has suggested that IR injury was attenuated by this polymeric compound in an animal model. The underlying mechanism could be elaborated via the reduction of neutrophil infiltration to alleviate tissue damage caused by IR injury (41) (**Figure 2**). In addition, ethyl acetate extract prevents myocardial IRI *in vivo* by inhibiting lipid peroxidation (42), and a recent study also showed that acetate ameliorated intestinal IRI as a novel therapeutic potential (43). In cerebral IR injury, carvacryl acetate provides neuroprotection against oxidative stress (44) (**Figure 2**). However, it is important to note that these results were obtained through experimental studies, and further research is needed to investigate their potential clinical applications.

Itaconate is a key immunometabolite produced by cis-aconitate from the TCA cycle in response to a variety of stimuli, such as aconitate decarboxylase 1 (ACOD1) in macrophages (45) (**Figure 1**). ACOD1 has been shown to suppress cerebral IR injury in mice (46). Itaconate exerts anti-inflammatory signaling by modulating macrophage metabolism and inhibiting SDH-mediated oxidation of succinate (47). Itaconate has been shown to reduce ROS production in activated M1 macrophages and is effective against cardiac, cerebral, and liver IR injuries (37, 48) (**Figure 2**). In the context of LT, targeting the itaconate pathway may provide an antioxidant response to inhibit liver damage from IR injury during LT (49). Hence, targeting itaconate will be considered a potential therapeutic strategy to protect the liver from IR injury and improve postoperative outcomes.

## 3 Targets in lipid metabolism

Dysregulation of lipid metabolism, which alters primary metabolic styles under metabolic reprogramming, has become a hallmark of recent cancer studies and may be associated with HCC development and progression. A better understanding of lipid metabolism and related signaling pathways may shed light on a therapeutic strategy to treat HCC by targeting lipid metabolism or metabolites to modulate the TME (50). Increasing evidence suggests that the accumulation of lipid metabolic products leads to tumor progression and local immunosuppression in the TME. Therefore, targeting lipid metabolism may be a potential therapeutic approach for the treatment of HCC. However, further research is needed to fully understand the role of lipid metabolism in HCC and its potential clinical applications.

### 3.1 Influence of SREBP-1 on HCC development

Sterol regulatory element-binding protein 1 (SREBP-1) is a ubiquitous transcription factor that activates the SREBP-1-mediated lipogenic pathway in HCC. Upregulation of SREBP-1 promotes the synthesis of fatty acids, increases cholesterol uptake into hepatocytes, and modulates mitochondrial fatty acid oxidation (FAO), according to a number of experimental studies (51–53). Moreover, high expression of the SREBP-1 protein is strongly associated with poor prognosis based on large-scale gene expression profiling (54). The suppression of SREBP-1 in HCC cells can lead to growth arrest and apoptosis, whereas overexpression of SREBP-1 promotes cell proliferation. These findings suggest that SREBP-1 could potentially be targeted for therapeutic purposes in the treatment of HCC (55) (**Figure 2**).

### 3.2 Role of miRNAs in the regulation of lipid metabolism and HCC progression

Furthermore, several microRNAs (miRNAs) mediate the pathogenesis of HCC by regulating lipid metabolism-related proteins (56–63) (**Figure 2**). For example, when miRNA1207-5p targets fatty acid synthase (FASN), it can inhibit HCC invasion by suppressing the protein kinase-B (Akt)/mTOR signaling pathway (57, 58). Conversely, when FASN is upregulated, it can reverse the inhibitory effects of miRNA-1207-5p on HCC cells (56, 64). Experimental research conducted by Wu et al. discovered a mechanism by which miRNA-21 contributes to the accumulation of lipids in the liver and the progression of HCC (58). This mechanism involves the interaction of miRNA-21 with the HBP1-p53-SREBP1c signaling pathway. Based on these findings, the miRNA-21-antisense oligonucleotide may be a potential therapeutic target for the treatment of HCC. In addition, miRNAs also play important roles in the regulation of lipid-metabolizing enzymes by interacting with metabolic-related transcription factors. Several miRNAs, such as miRNA-33a/b, miRNA-182, miRNA-96, and miRNA-24, have been identified as participating in the regulation of SREBPs (61–63). Studies have shown that miRNA-631 and miRNA-155 have a negative regulatory effect on liver X receptor alpha (LXRα) (65, 66). LXRα plays a crucial role in the regulation of fatty acid metabolism by controlling SREBP1-c and downstream targets involved in fatty acid synthesis (67, 68).

### 3.3 Fatty acids transport and uptake

Downregulation of fatty acid transport protein 2 (FATP2) by knockdown or genetic deletion of FATP5 has been shown to decrease fatty acid uptake, which may have potential as a new strategy for HCC (50, 65, 69) (**Figure 2**). In clinical research, the expression of carnitine palmitoyltransferase 2 (CPT2), which is responsible for converting acylcarnitine back to acyl-CoA, is reduced in human steatohepatitic HCC (SH-HCC) (**Figure 2**). This leads to a significant accumulation of acylcarnitine species that can be detected in the serum (66). This suggests that measuring serum acylcarnitine levels may serve as a potential biomarker for HCC. More importantly, inhibition of the FAO pathway can be achieved not only by downregulation of CPT2 but also by changes in peroxisome proliferator-activated receptor (PPAR) activation (66, 70). The altered expression of PPARs mainly induces mitochondrial metabolic dysfunction, which can suppress FAO, accumulate ROS that may promote cancer cell growth, and promote lipogenesis (70) (**Figure 2**).

### 3.4 Lipid metabolic reprogramming between immune and HCC cells

The immune responses of immune cells must change and adapt to the TME. Understanding the significant role of tumor immunometabolism reprogramming suggests a crucial targeting signal between immune cells and tumor cells, but more exploration is needed on the complex crosstalk between liver cancer cells (71). In TAMs, changes in their lipid profile are frequent. Yeung et al. (72) concluded that interactions between TAMs and HCC cells *in vitro* promoted tumor cell migration through M2 monocyte-derived macrophages (MDMs) in an FAO-dependent manner under IL-1β secretion (**Figure 2**). As for effector T cells, their activities are restricted in the TME according to recent studies (73, 74). Tumor-associated dendritic cells (TADCs) express the scavenger receptor-like macrophage scavenger receptor 1 (MSR1) to facilitate lipid uptake and accumulation in HCC (75). Increased cholesterol facilitates the antitumor effects of natural killer cells (NKs) and blocks tumor growth *in vivo* (76) (**Figure 2**). In short, future research should focus on emphasizing the underlying mechanisms of lipid metabolism in the HCC immune microenvironment.

### 3.5 Rapamycin and mTOR targeting lipid metabolism

The mTOR complex is a critical regulator of lipid metabolism and contains two distinct complexes: mTOR complex 1 (mTORC1) and mTOR complex 2 (mTORC2) (77, 78). Lipid metabolism is mainly controlled by mTORC1, whereas liver tumorigenesis is regulated by mTORC2 (77, 79). Therefore, many drugs targeting lipid metabolism have been developed for patients with HCC. Rapamycin, an immunosuppressant that inhibits mTOR, is widely used post-LT to regulate lipid metabolism (79, 80) (**Figure 2**). Rapamycin has shown anticancer potential in the treatment of HCC progression (81). Due to the synthesis and accumulation of lipids mediated by the mTOR pathway, rapamycin may cause lipid metabolism disorders after transplantation. On the one hand, rapamycin inhibits lipid storage in adipose tissue (82–86). On the other hand, it can cause lipid accumulation in the blood and liver, according to clinical data (87–90). In summary, the mTOR pathway may be a potential target to regulate immune cells by manipulating cellular lipid metabolism. Inhibition of mTOR with rapamycin can block the development of macrophages and CD4^+^ T cells in liver cancer (91, 92). Therefore, future studies should focus on combining immunity and lipid metabolism to develop novel therapeutic methods that may benefit patients with HCC.

## 4 Targeting glutamine metabolism

In the context of glutamine metabolism, the accumulation of succinate in lipopolysaccharide (LPS)-activated macrophages facilitates the anaplerosis of alpha-ketoglutarate (α-KG) into the TCA cycle (93) (**Figure 1**). Based on a number of experimental results, glutamine, as a central anabolic metabolite in the TCA cycle, is used more extensively in M2 metabolism than in M1 macrophages (94, 95). Glutamine is also essential for T cell survival and proliferation. Reduced glutamine and α-KG levels suggest that metabolites from these metabolic pathways may enhance Treg cell function (96). Glutaminolysis pathways have garnered considerable attention as immune cell activation and differentiation alter metabolic patterns, with reprogramming dependent on glycolysis in the TME.

### 4.1 Protein metabolism-directed drug development

Reversal of rejection after solid organ transplantation *in vivo* has been achieved by using a combination of 6-diazo-5-oxo-L-norleucine (DON), a glutamine metabolism inhibitor (97) (**Figure 2**). Based on experimental data, Lee et al. have shown that targeting glutamine metabolism promotes immune tolerance and leads to a good prognosis (97). Aerobic glycolysis is of crucial importance in the occurrence and development of tumors. 2-deoxy-D-glucose (2-DG), a glycolytic inhibitor, can selectively damage effector T cells by inhibiting hexokinase, which is involved in glycolysis (**Figure 2**). Blocking glycolysis in combination with DON and metformin [an oral hypoglycemic agent for type 2 diabetes mellitus that activates adenosine monophosphate (AMP)-activated protein kinase (AMPK), inhibits mitochondrial respiratory complex I of the electron transport chain (ETC), and promotes FAO] prevents graft rejection in solid allograft transplantation models (98) (**Figure 2**). It is worth noting that metformin attenuates ischemia-reperfusion (IR) injury in fatty liver disease via the toll-like receptor 4 (TLR4)/NF-κB axis (99). Preconditioning with metformin also lowers hepatobiliary injury and improves hepatobiliary function in an *in situ* and *ex-situ* model of rat donor LT (100). Chen et al. have shown that methylcrotonoyl-CoA carboxylase 2 (MCCC2) correlates with the leucine metabolism pathway in the progression of HCC and may be a new target for HCC (101) (**Figure 2**). The branched-chain amino acids to tyrosine ratio can predict the prognosis of HCC treatment based on clinical practice data (102). Solute carrier family 27 member 5 (SLC27A5) and tyrosine metabolizing enzymes identified by Wang et al. coordinate lipid and tyrosine metabolism in HCC, alter the tumor cell cycle, and are potential targets for cancer treatment (103) (**Figure 2**).

## 5 Conclusions and perspectives

In recent years, numerous studies have investigated changes in metabolites during immunometabolic processes during HCC progression and after LT. The successful application of immunometabolism modulation is demonstrated by the FDA’s approval of small molecule metabolites for the treatment of specific tumors (104). Furthermore, several other metabolites are currently in clinical trials and awaiting approval (NCT04471415, NCT03272256, and NCT03179904). However, the underlying modulatory network of metabolites in HCC cells in the TME remains unclear. The potential effects of these drugs on the hepatic TME are unknown, and this review does not comprehensively cover all metabolites targeting immunometabolism, as this is an emerging field that holds promise for new tumor-metabolizing drugs. Further extensive research is necessary to examine how metabolic drugs affect the hepatic TME, which is complex and varies with the composition of different cells that interact with HCC cells. The composition of the immune microenvironment also differs before and after LT, and targeted immunometabolism therapy requires the identification of synergistic targets in liver cancer cells and immune cells to achieve synergistic effects. Therefore, it is fundamental to investigate how targeting various metabolites and metabolic enzymes affects T-cell function.

Although numerous metabolites have been investigated for their potential as tumor-targeted therapies, the responsiveness of specific tumor types to inhibitors, single agents, or combinations with other therapies has not yet been fully clarified. However, the targeting of specific metabolites and the flexibility of tumor immunometabolism will also present significant challenges. In the years ahead, clinical trials should be conducted to explore metabolites as a potential avenue for further research. For example, a polymeric compound that specifically targets succinate, which has been shown to play a regulatory role in cancer-immune cross-talk, can reduce tumor cells in mice. Clearly, more attention should be paid to targeting miRNAs for the treatment of HCC with abnormal lipid metabolism (105–107). Irrespective of future developments, the optimal outcome is to target tumor immunometabolism while simultaneously enhancing antitumor immunity in a synergistic manner.

In conclusion, additional studies examining metabolites derived from carbohydrate, lipid, and protein metabolism are needed to identify novel therapeutic targets in the HCC setting and post-transplantation. Future investigations should strive to integrate immunity and metabolism to develop innovative therapeutic strategies that target specific metabolites and elucidate their detailed mechanisms. Ultimately, these efforts have the potential to provide considerable benefit to patients with HCC.

## Author contributions

WZ designed the study and wrote the manuscript. YZ wrote the manuscript. WZ and RL prepared the figures and revised the manuscript. QH and RL supervised the study. All authors contributed to the article and approved the submitted version.
